# Transfer RNA methyltransferases with a SpoU-TrmD (SPOUT) fold and their modified nucleosides in tRNA

**DOI:** 10.3390/biom7010023

**Published:** 2017-02-28

**Authors:** Hiroyuki Hori

**Affiliations:** Department of Materials Science and Biotechnology, Graduate School of Science and Engineering, Ehime University, 3 Bunkyo-cho, Matsuyama, Ehime 790-8577, Japan; hori@eng.ehime-u.ac.jp; Tel.: +81-89-927-8548; Fax: +81-89-927-9941

**Keywords:** knot, methyltransferase, SpoU-TrmD, RNA modification, tRNA

## Abstract

The existence of SpoU-TrmD (SPOUT) RNA methyltransferase superfamily was first predicted by bioinformatics. SpoU is the previous name of TrmH, which catalyzes the 2’-*O*-methylation of ribose of G18 in tRNA; TrmD catalyzes the formation of *N*^1^-methylguanosine at position 37 in tRNA. Although SpoU (TrmH) and TrmD were originally considered to be unrelated, the bioinformatics study suggested that they might share a common evolution origin and form a single superfamily. The common feature of SPOUT RNA methyltransferases is the formation of a deep trefoil knot in the catalytic domain. In the past decade, the SPOUT RNA methyltransferase superfamily has grown; furthermore, knowledge concerning the functions of their modified nucleosides in tRNA has also increased. Some enzymes are potential targets in the design of anti-bacterial drugs. In humans, defects in some genes may be related to carcinogenesis. In this review, recent findings on the tRNA methyltransferases with a SPOUT fold and their methylated nucleosides in tRNA, including classification of tRNA methyltransferases with a SPOUT fold; knot structures, domain arrangements, subunit structures and reaction mechanisms; tRNA recognition mechanisms, and functions of modified nucleosides synthesized by this superfamily, are summarized. Lastly, the future perspective for studies on tRNA modification enzymes are considered.

## 1. Introduction

**Figure 1:**
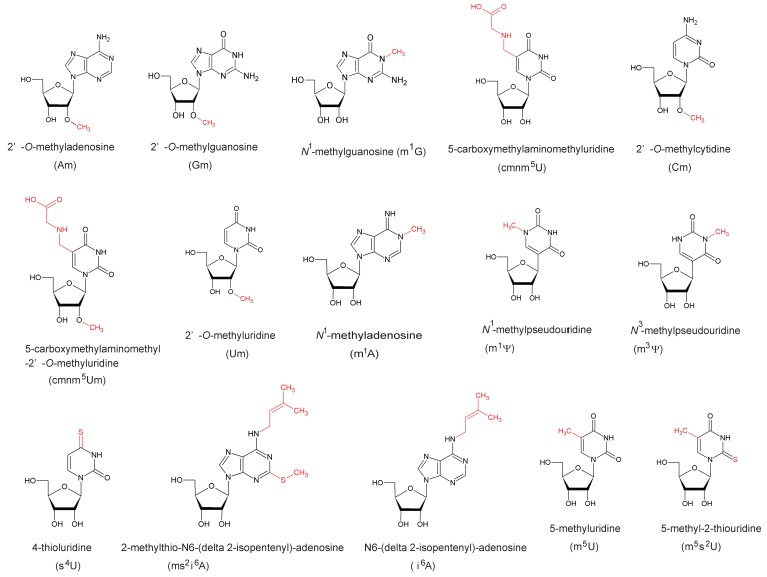
Structures and abbreviated names of the modified nucleosides described in this review. The modifications are colored in red.

**Figure 2:**
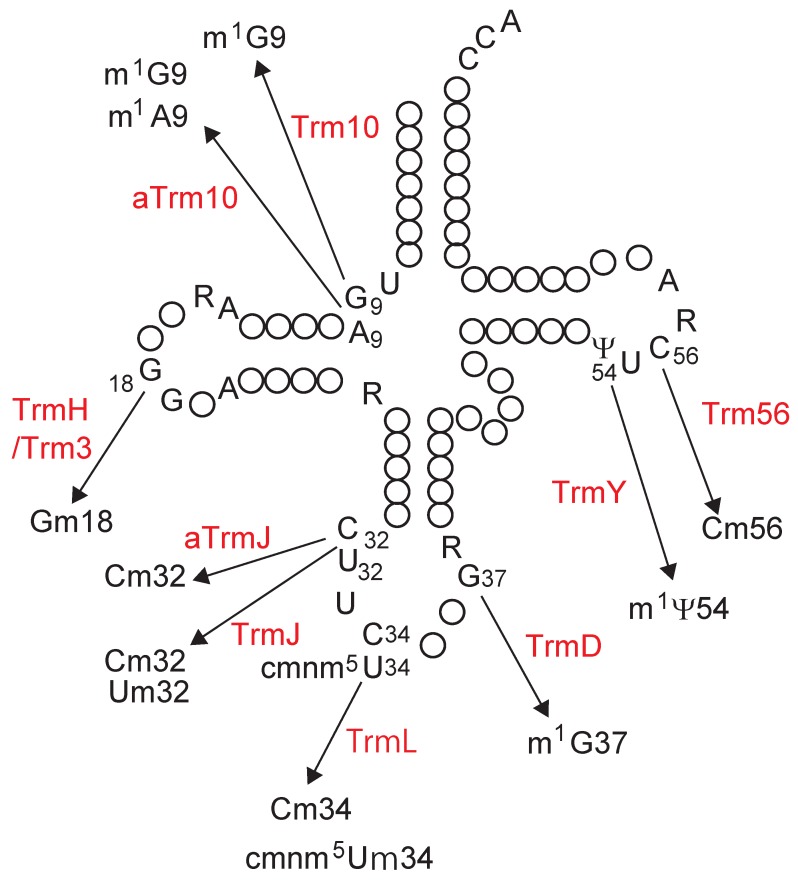
Modification positions in tRNA and the responsible SpoU-TrmD (SPOUT) tRNA methyltransferases. The secondary structure of tRNA is represented in cloverleaf structure. The conserved nucleotides in tRNA are depicted as follows: adenosine, A; guanosine, G; cytidine, C; uridine, U; purine, R; pseudouridine, Ψ. The modified positions are numbered and the associated enzymes are indicated in red. The structures of modified nucleosides and abbreviations of their names are shown in Figure 1.

The existence of the SpoU-TrmD (SPOUT) RNA methyltransferase superfamily was first predicted by bioinformatics studies [1,2,3]. The gene name, *spoU*, derives from an open reading frame for a protein of unknown function in the *Escherichia coli* *spoT* operon. In 1993, Koonin and Rudd predicted the *spoU* gene product to be an RNA methyltransferase based on the similarity of its deduced amino acid sequence to that of *Streptomyces azureus* Tsr [1], which catalyzes methylation of the ribose of adenosine (A) at position 1067 in 23S rRNA to form 2’-*O*-methyladenosine (Am1067) [4]. The structures of modified nucleosides covered in this review together with their positions in tRNA and the responsible enzymes are summarized in Figure 1 and Figure 2, respectively: the structures of modified nucleosides are presented according to the MODOMICS database (http://modomics.genesilico.pl/) [5].

**Figure 3:**
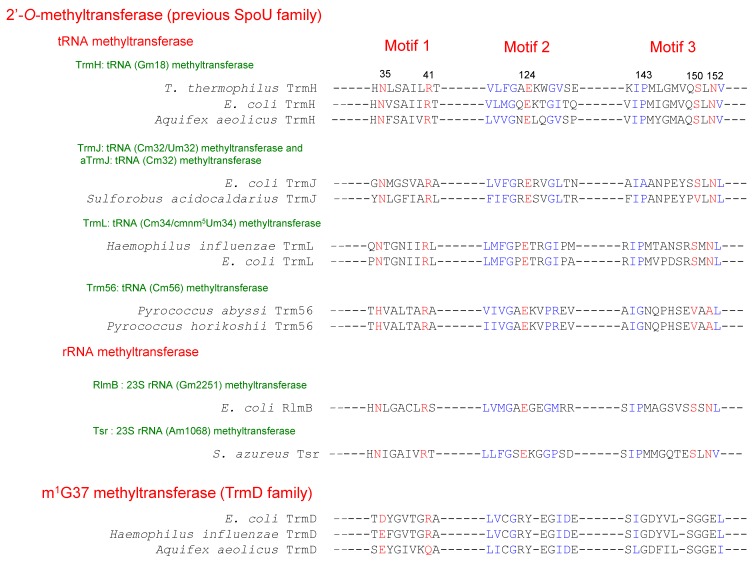
The conserved motifs in the TrmH (SpoU) and TrmD families. The amino acid sequence alignment is based on the reference [3], and has been modified in accordance with biochemical data. Color (blue and red) letters indicate the conserved amino acid residues, reported by Anantharaman et al. [3]. Red letters indicate the amino acid residues that are essential for the methyl-transfer reaction by *Thermus thermophilus* TrmH. Numbers indicate the positions of amino acid residues in *T. thermophilus* TrmH. *T. thermophilus*: *Thermus thermophilus*; *E. coli*: *Eshcerichia coli*; *S. aureus*: *Streptomyces aureus*.

In 1996, Gustafsson et al. [2] compared the amino acid sequences of *E. coli* SpoU, *S. azureus* Tsr and yeast Pet56, which catalyzes methylation of the ribose of guanosine (G) at position 2270 in mitochondrial 21S rRNA to form 2’-*O*-methylguanosine (Gm2270) [6]. They reported that the three RNA 2’-*O*-methyltransferases have three conserved motifs, termed motifs 1, 2 and 3 (Figure 3) [2]. Almost simultaneously, analysis of an *E. coli spoU* gene deletion strain revealed that *spoU* encodes a tRNA (Gm18) methyltransferase [7], which catalyzes the transfer of a methyl group from *S*-adenosyl-l-methionine (AdoMet) to the 2’-OH of ribose of G18 in tRNA to produce Gm18 modification (Figure 1 and Figure 2) [8]. According to the nomenclature of tRNA methyltransferase genes, the gene name, *spoU* was subsequently altered to *trmH* (as the eighth identified gene for tRNA methylation in bacteria) [7] and *spoU* remains only in the name of the superfamily. In 2002, Anantharaman et al. reported that SpoU (TrmH) family members share amino acid sequence homologies with TrmD family members (Figure 3) [3]. TrmD catalyzes the formation of *N*^1^-methylguanosine at position 37 (m^1^G37) in tRNA (Figure 1 and Figure 2) [9]. Thus, although the SpoU (TrmH) and TrmD families were originally considered to be unrelated, this bioinformatics study suggested that they might share a common evolution origin and form a single superfamily [3].

**Figure 4:**
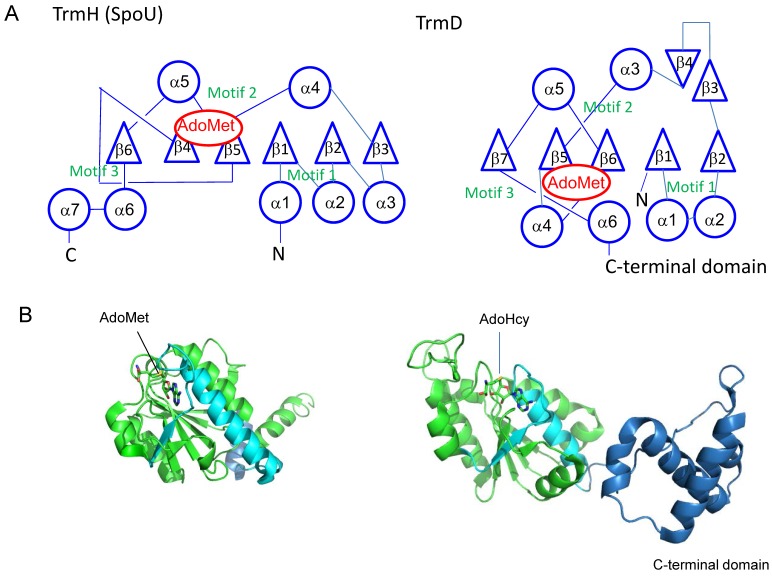
(**A**) Topological knot structures in TrmH (SpoU) and TrmD. The representations of topologies are in accordance with the references [10,15,19]. Circles, triangles and *S*-adenosyl-l-methionine (AdoMet) indicate α-helices, β-strands and AdoMet binding site, respectively. (**B**) Cartoon models of *T. thermophilus* TrmH (Protein Data Bank ID: 1v2x) and *Escherichia coli* TrmD (Protein Data Bank ID: 1p9p) subunits. To show the knot structures, the C-terminal regions of catalytic domains are colored in cyan. The C-terminal domain of *E. coli* TrmD is indicated in blue. The bound AdoMet and *S*-adenosyl-l-homocysteine (AdoHcy) are shown by stick models.

The common feature of SPOUT RNA methyltransferases is the formation of a deep trefoil (topological) knot in the catalytic domain (Figure 4). In 2002, Nureki et al. reported that a hypothetical RNA 2’-*O*-methyltransferase (RrmA) from *Thermus thermophilus* has a deep trefoil knot, which is formed by the threading of a polypeptide chain through a loop [10]. In addition, Michel et al. have reported that *E. coli* RlmB, a eubacterial counterpart of Pet56 [11], has a knot region in its C-terminal catalytic domain [12]. In 2003, Lim et al. reported that YibK from *Haemophilus influenzae* has a knot structure, which forms an *S*-adenosyl-l-homocysteine (AdoHcy)-binding site [13]. AdoHcy is derived from AdoMet via a methyltransfer reaction. Later, the enzymatic activity of YibK was identified as a tRNA methyltransferase: *E. coli* YibK was shown to catalyze the 2’-*O*-methylation of both cytidine at position 34 (C34) and 5-carboxymethylaminomethyluridine at position 34 (cmnm^5^U34) in tRNA^Leu^ isoacceptors, to form 2’-*O*-methylcytidine (Cm34) and 5-carboxymethylaminomethyl-2’-*O*-methyluridine (cmnm^5^Um34), respectively (Figure 1 and Figure 2) [14]. As a result, thename of YibK was altered to TrmL [14]. In 2003, three groups independently solved the crystal structures of TrmD, and reported that the TrmD also has a trefoil knot structure (Figure 4) [15,16,17]. In 2004, our group solved the crystal structure of *T. thermophilus* TrmH (SpoU) and found that TrmH also has a deep trefoil knot (Figure 4) [18]. Figure 4A and B show the topologies and subunit structures of TrmH (SpouU) (left) and TrmD (right), respectively. Together, these bioinformatics and crystal structure studies established the basic concept of the SPOUT RNA methyltransferase superfamily.

AdoMet-dependent enzymes can be divided into more than five classes according to the structure of their catalytic domain [20]. Most methyltransferases have a Rossman fold catalytic domain and are classified as class I enzymes. In contrast, members of SPOUT RNA methyltransferase superfamily are classified as class IV enzymes, whose catalytic domain forms a deep trefoil (topological) knot. Recently, new folds in the catalytic domains of RNA methyltransferases have been reported [21]. Furthermore, radical *S*-adenosyl-l-methionine (SAM) dependent-methyltransferase have been discovered [22,23,24,25,26]. These latter AdoMet-dependent methyltransferases are likely to be categorized into new classes.

For a decade, the SPOUT RNA methyltransferase superfamily has been growing in number, and knowledge concerning the functions of their modified nucleosides has increased. Moreover, in addition to the RNA methyltransferase function, a protein methyltransferase [27] and an RNA aminocarboxypropyl-transferase [28] with SPOUT folds have been discovered. Although the SPOUT fold has been extended to these other enzymes, in this review, tRNA methyltransferases in the SPOUT RNA methyltransferase superfamily and their modified nucleosides in tRNA are focused owing to space limitation.

## 2. Classification of tRNA Methyltransferases with a SPOUT Fold

**Figure 5:**
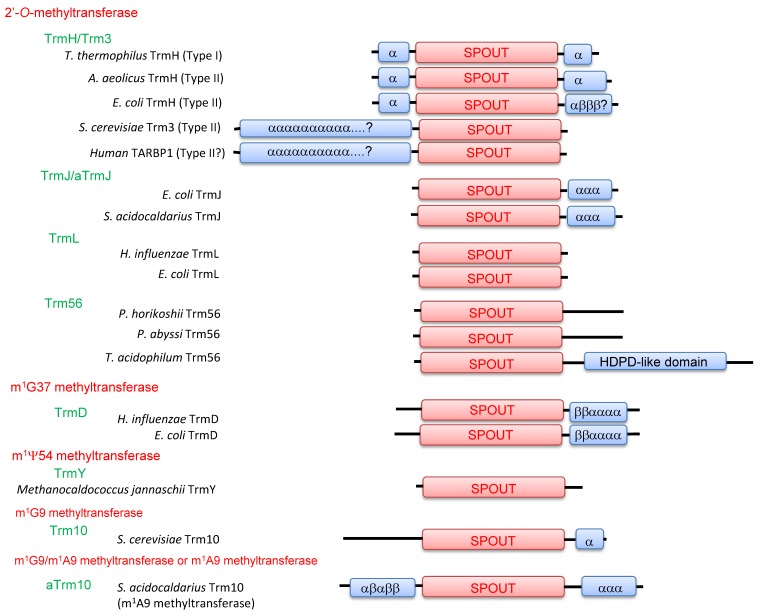
Domain structures of tRNA methyltransferases with a SPOUT fold. This figure is based on that by Tkaczuk et al. [29] and has been modified by data from recent crystal structure studies. The catalytic domain with a SPOUT fold is represented as “SPOUT”. “α” and “β” represent α-helices and β-strands, respectively. The HDPD-like domain indicates the His-Asp phosphodiesterase-like domain. *S. cerevisiae*: *Saccharomyces cerevisiae*; *H. influenzae*: *Haemophilus influenzae*; *P. horikoshii*: *Pyrococcus horikoshii*; *P. abyssi*: *Pyrococcus abyssi*; *T. acidophilum*: *Thermoplasma acidophilum*; *S. acidocaldarius*: *Sulfolobus acidocaldarius*.

The SPOUT RNA methyltransferase superfamily was established through the amino acid sequence motifs and the presence of a topological knot in the catalytic domain. Therefore, the protein families and subfamilies in this superfamily should be classified by differences in the structure of the catalytic domain and/or the catalytic mechanism. However, a more convenient way to classify them is based on the synthesized modified nucleoside and its position in tRNA (Figure 2 and Figure 5) because the reactivity of the target atom in the nucleoside and the structure of the modification site in the tRNA reflect the structure of the catalytic pocket, the catalytic mechanism, and the arrangement of domains and/or subunit structures.

Accordingly, tRNA methyltransferases with a SPOUT fold can be divided into five categories; 2’-*O*-methyltransferases, m^1^G37 methyltransferases, m^1^Ψ54 methyltransferases, m^1^G9 methyltransferases and m^1^A9/m^1^G9 methyltransferases (or m^1^A9 methyltransferases) (Figure 5). 2’-*O*-methyltransferases were previously categorized collectively as the SpoU (TrmH) family [3]. Indeed, three motifs are highly conserved among 2’-*O*-methyltransferases (Figure 3). In this review, however, 2’-*O*-methyltransferases are separated into the TrmH, TrmJ, TrmL and Trm56 families because their functions have been clarified (Figure 2) and they belong to different clusters of orthologous groups (COGs) [29].

Transfer RNA methyltransferases with a SPOUT fold have been reported from the three domains of life. In this review, the archaeal TrmJ and Trm10 enzymes are abbreviated as aTrmJ and aTrm10, respectively. Notably, aTrm10 and Trm10 families seem to be distinguished as independent enzyme families, because a recent crystal structure study has revealed that the catalytic amino acid residues in aTrm10 are different from those in Trm10 [30].

Lastly, RlmH, a methyltransferase for formation of 3-methylpseudouridine at position 1915 (m^3^Ψ1915) in 23S rRNA [31,32], has a subunit comprising only the SPOUT catalytic domain with conserved amino acid sequences related to those of the TrmD family [31]. Because m^3^Ψ modification has not yet been found in tRNA [33], details of RlmH are not described in this review.

## 3. Knot Structures, Domain Arrangements, Subunit Structures and Reaction Mechanisms of tRNA Methyltransferases with a SPOUT Fold

A knotted structure in proteins has been found not only in the SPOUT fold but also in other protein folds [34]. Clarification of the folding mechanism of a knotted structure in a polypeptide is one of the important issues in protein chemistry [35] and is also important for physiology. For example, a knotted protein cannot pass through mitochondrial pores without unfolding [36].

Among members of the SPOUT superfamily, TrmL and RlmH are minimalist proteins comprising only a SPOUT fold. Therefore, TrmL and RlmH have been used as model proteins for the investigation of knot formation [37]. The knotted structure in TrmL spontaneously refolds from the denatured form [38]. A similar phenomenon has also been observed in the case of TrmH. *Thermus thermophilus* TrmH binds tightly to AdoHcy-Sepharose 4B and can be eluted by 6 M urea; however, the activity of TrmH is recovered by dialysis [39]. Indeed, we used this purification method for the preparation of TrmH, and the crystalized TrmH had a trefoil-knot structure [18]. Thus, although *T. thermophilus* TrmH has N- and C-terminal extensions (Figure 5), the knotted structure can be refolded spontaneously. Later, this purification method brought us unexpected benefits: intrinsic AdoMet and AdoHcy, which were bound in the AdoMet-binding pocket, were removed from the purified enzyme through AdoHcy-affinity column chromatography. Therefore, the purified TrmH protein was useful for pre-steady state kinetic studies [40,41].

In general, the C-terminal region of a tRNA methyltransferase with a SPOUT fold is short (Figure 5). This observation suggests that there are limitations to the length and sequence of C-terminal polypeptide that can be threaded through a polypeptide loop during the formation of a knotted structure. Mallam et al. have reported interesting experimental results [42]. They fused a ThiS protein (91 amino acids) to the N- and/or C-terminal region(s) of TrmL and RlmH. The fusion proteins formed dimer structures and AdoHcy was bound to them, indicating that they correctly formed the knotted structure even though the ThiS protein was fused at their C-terminal region [42]. Thus, a polypeptide of considerable size can be threaded through the loop in the SPOUT fold.

A similar phenomenon was observed in the case of *Thermoplasma acidophilum* Trm56 (Figure 5) [43]. A bioinformatics study has predicted that this protein has a His-Asp phosphodiesterase (HDPD)-like domain at its C-terminal region [29]. The purified protein showed Trm56 (tRNA (Cm56) methyltransferase) activity [43], strongly suggesting that the catalytic domain of *T. acidophilum* Trm56 forms a SPOUT fold even though the protein has a polypeptide region of 130 amino acids at the C-terminus.

In addition, following findings concerning the knot structure in the SPOUT RNA methyltransferases have been reported. In the case of *E. coli* TrmD, sliding of the knot towards the C-terminus has been observed during the denaturation process [44]. Furthermore, AdoMet binding induces internal movement in the knot of TrmD [45]. Although the knotted structure can be formed spontaneously, the presence of molecular chaperones accelerates the folding [46].

Members of tRNA methyltransferases with a SPOUT fold have a short N-terminal region as compared with rRNA methyltransferases. One exception is Trm3 (Figure 5) [47], a eukaryotic counterpart of TrmH. Trm3 possess a long N-terminal region, which is predicted to form α-helices and likely to localize at the nuclear membrane. The human homolog of Trm3 is TARBP1 and the structure of C-terminal SPOUT fold region has been reported [48]. So far, however, the enzymatic activity of human TARBP1 has not been reported.

For a decade, a dimer structure has been believed to be essential for the enzymatic activity of tRNA methyltransferases with a SPOUT fold because the catalytic pocket is formed by the interaction of two subunits. Indeed, TrmH, TrmJ, TrmL, Trm56, TrmD and TrmY form a dimer structure. Recently, however, it was reported that Trm10 and aTrm10 are monomeric enzymes [30,49]; thus, catalytic pockets of Trm10 and aTrm10 are formed in one subunit.

**Figure 6:**
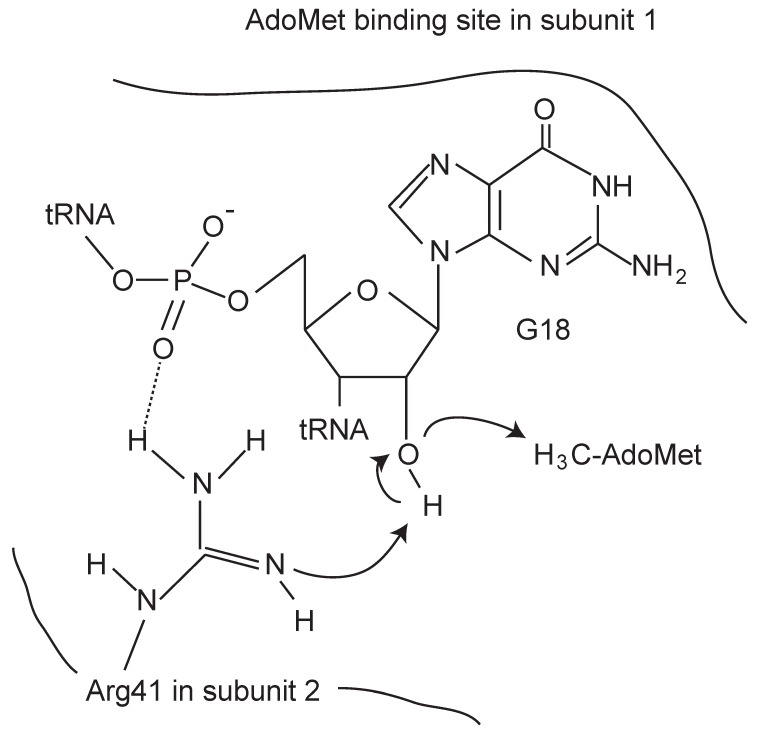
Schematic drawing of the hypothetical catalytic mechanism of *T. thermophilus* TrmH.

The reaction mechanisms of base methylations by tRNA methyltransferases have been recently reviewed [50,51]. To avoid duplication, therefore, I describe only the 2’-*O*-methylation of ribose by tRNA methyltransferases with a SPOUT fold. A structure-based site-directed mutagenesis study of *T. thermophilus* TrmH elucidated the importance of several amino acid residues in the methyltransfer reaction such as Asn35, Arg41, Glu124, Ser150 and Asn152 [18,52] (indicated in red in Figure 3). The high conservation of these amino acid residues in three motifs among the 2’-*O*-methyltransferases suggests that the enzymes have a common catalytic mechanism. Substitution of these residues by other amino acids leads to near-complete loss of TrmH enzymatic activity. We proposed the following hypothetical catalytic mechanism, in which Arg41 in *T. thermophilus* TrmH is the catalytic center (Figure 6). *T. thermophilus* TrmH is a dimer enzyme [18]: one subunit functions as the AdoMet-binding site while Arg41 in the other subunit (tRNA-binding subunit) is activated by phosphate in tRNA and causes deprotonation of the 2’-OH of ribose at position 18 in tRNA. The deprotonated oxygen atom leads to nucleophilic attack the methyl group of AdoMet. The importance of the corresponding Arg residues in Trm56 [53] and TrmL [54] in the catalytic mechanism has been also reported. Thus, data from other 2’-*O*-methyltransferases are consistent with our hypothetical mechanism. Substitution of Glu124 in *T. thermophilus* TrmH by Ala or Gln caused loss of enzymatic activity and loss of affinity for tRNA [52]. In the case of Trm56, however, substitution of corresponding Glu residue by Ala did not abolish enzymatic activity [53]. This difference might be caused by the different target sites of TrmH and Trm56 in tRNA. Furthermore, the conserved Asn35 residue in *T. thermophilus* TrmH is involved in the release of AdoHcy [52]. To clarify the precise catalytic mechanism of 2’-*O*-methyltransferases, a crystal structure study of the enzyme-tRNA complex is necessary.

## 4. Transfer RNA Recognition Mechanism

Transfer RNA methyltransferases with a SPOUT fold possess a seemingly similar catalytic domain. Nevertheless, the enzymes produce different modified nucleotides at various positions in tRNA. In some cases (for example, TrmD), the enzyme modifies only specific tRNAs. Furthermore, the target site for methylation is often embedded in the l-shaped tRNA structure. Consequently, in many cases, recognition of tRNA by tRNA methyltransferases seems to involve multiple steps (namely, initial binding and induced-fit processes). Clarifying the mechanism of recognition of the target site in a specific nucleic acid is one of the important topics in studies on nucleic acid-related enzymes. In this section, knowledge of the tRNA recognition mechanisms of tRNA methyltransferases with a SPOUT fold are collated.

### 4.1. TrmH and Trm3

TrmH produces a Gm18 modification in tRNA as described above [7,8,39]. TrmH can be divided into two types based on their tRNA specificity. Type I enzymes, such as *T. thermophilus* TrmH, methylate all tRNA species [55], whereas Type II enzymes, including *E. coli* [7] and *A. aeolicus* TrmH [56,57], methylate specific tRNAs. Indeed, we determined the RNA sequence of *A. aeolicus* tRNA^Cys^ and found that it did not contain Gm18 modification [58], consistent with in vitro methylation experiments [56]. TrmH from *T. thermophilus* has been shown to methylate a truncated tRNA fragment (5’-half fragment) [59] and ultraviolet (UV)-induced crosslinking of 4-thiouridine at position 8 (s^4^U8) in substrate tRNA decreased the velocity of methylation by *T. thermophilus* TrmH [60]. The l-shaped structure of tRNA and the presence of conserved nucleotides in tRNA were found to accelerate the initial velocity of methyltransfer reaction by *T. thermophilus* TrmH [55,61].

A structure-based site-directed mutagenesis study of *T. thermophilus* TrmH [62] revealed that the conserved basic amino acid residues can be categorized according to their role (i) in the catalytic center (Arg41) (Figure 6); (ii) in the initial site of tRNA binding (Lys90, Arg166, Arg168, and Arg176); (iii) in the tRNA binding site required for continuation the catalytic cycle (Arg8, Arg19, and Lys32); (iv) in the structural element involved in release of AdoHcy (Arg-11-His-71-Met-147 interaction); (v) in the assisted phosphate binding site (His34); or (vi) in an unknown function (Arg109). A stopped-flow pre-steady state kinetic analysis showed that the binding of TrmH to tRNA is composed of at least three steps, an initial bi-molecular binding and two subsequent uni-molecular induced-fit processes [40]. Furthermore, TrmH methylates guanosine in d-loops ranging from four to 12 nucleotides in length, which suggests that selection of the position of guanosine within the d-loop is relatively flexible [40]. Pre-steady state kinetic analysis of complex formation between mutant TrmH proteins and tRNA by stopped-flow fluorescence measurement revealed that the C-terminal region acts in the initial binding process, during which non-substrate tRNA is not excluded, whereas structural movement of the motif-2 region of the catalytic domain in an induced-fit process is involved in substrate tRNA discrimination [41].

In *E. coli*, only 14 of the 47 tRNA species possess Gm18 modification [33]. The substrate tRNA selection mechanism of *E. coli* TrmH has not been clarified. Comparison of amino acid sequences of TrmH enzymes, limited proteolysis [39], crystal structures of TrmH enzymes [18,57] and site-directed mutagenesis [41,52,62] strongly suggested that the C-terminal region in *E. coli* TrmH is involved in substrate selectivity. To confirm this idea, we made chimeric enzymes of TrmH from *E. coli* and *T. thermophilus* and tested their substrate selectivity. Contrary to our expectation, the substrate tRNA was selected by not the C-terminal region of *E. coli* TrmH but the catalytic domain [41]. Taking these observations together, the C-terminal region of TrmH is involved in the initial binding process, during which substrate and non-substrate tRNAs are not distinguished. Non-substrate tRNA may be released from the *E. coli* TrmH-tRNA complex during the structural change process.

The eukaryotic counterpart of TrmH is Trm3 and yeast Trm3 has been shown to require the tertiary base pairs in the l-shaped tRNA for effective Gm18 formation [47]. The mechanism of substrate tRNA selection by Trm3 is unknown.

### 4.2. TrmJ and aTrmJ

TrmJ [63] and aTrmJ [64] catalyze the methylation of ribose at position 32 in eubacterial and archaeal tRNA, respectively. *E. coli* TrmJ requires interaction with the t- and d-loops in tRNA for methylation [64,65]. Given that deletion of the aminoacyl-stem leads to loss of the methyl group acceptance activity of tRNA^Met^ [65], the positive determinants for *E. coli* TrmJ must be included in the aminoacyl-stem and anticodon-arm. Although only Cm32 and Um32 modifications are observed in *E. coli* tRNA, *E. coli* TrmJ can produce Am32 and Gm32 in mutant tRNA^Ser^ transcripts [64]. Thus, the base at position 32 is not recognized by *E. coli* TrmJ.

In contrast, aTrmJ (*Sulfolobus acidocaldarious* TrmJ) can modify stem-loop RNA corresponding to the anticodon-arm and methylate the ribose of only cytidine at position 32 (C32) [64]. Thus, aTrmJ recognizes the cytidine base at position 32. Although the amino acid sequences and overall structures of TrmJ and aTrmJ resemble each other, the positively charged area at the cleft of the dimer interface in the catalytic domain of *E. coli* TrmJ is wider than that of aTrmJ [64]. This difference might be related to variations in the recognition of tRNA by TrmJ and aTrmJ.

The crystal structures of the catalytic domain and C-terminal region of *E. coli* TrmJ were reported in 2015 [65]. A structure-based site-directed mutagenesis study revealed that Arg82 and Arg84 residues in a TrmJ-specific motif (TXARXR sequence) and an α8-helix in the C-terminal region of *E. coli* TrmJ are important for tRNA recognition [65]. During the preparation of this manuscript, characterization of *Pseudomonas aeruginosa* TrmJ, together with its crystal structure, was reported [66]. In vivo and in vitro experiments revealed that A32 in *P. aeruginosa* tRNA^Pro^_GGG_ was modified to Am32 by TrmJ; however, C32 in tRNA^Ser^_UGA_ was not methylated by *P. aeruginosa* TrmJ, showing that the substrate tRNA species for *P. aeruginosa* TrmJ is different from those for *E. coli* TrmJ.

### 4.3. TrmL

The xm^5^U modification at the wobble position of an anticodon in tRNA is complex and is synthesized by multiple steps [67,68]. In the case of *E. coli*, a portion of cmnm^5^U34 in tRNA^Leu^_cnmn_^5^_UAA_ and C34 in tRNA^Leu^_CAA_ is modified to cmnm^5^Um34 and Cm34, respectively, by TrmL [14]. The crystal structure of TrmL (previously called YibK) was reported in 2003 [13], and TrmL has been used as a model protein with a SPOUT fold [36,37]. For a long time, however, the enzymatic activity of TrmL was not identified because it does not methylate unmodified tRNA transcripts.

The enzymatic activity of TrmL was detected only by using *E. coli trmL* (*yibK*) gene disruptant strain [14]. Experiments with an *E. coli trmL* and *miaA* double-knock-out strain revealed that 2-methylthio-*N*^6^-(Δ^2^-isopentenyl)-adenosine at position 37 (ms^2^i^6^A37) (Figure 1), which is synthesized by multiple steps including the reaction by MiaA (tRNA Δ^2^-isopentenylpyrophospate transferase [69]), is essential for tRNA recognition by TrmL [14]. A TrmL-tRNA docking model based on the crystal structure and a site-directed mutagenesis study suggested that arginine residues in one subunit capture the anticodon-stem, while another subunit functions as the AdoMet-binding subunit [54]. This model suggested a possibility of direct interaction between TrmL and ms^2^i^6^A37 in tRNA [54]. In 2015, it was reported that chemically synthesized stem-loop RNA containing Ψ32, i^6^A37 and Ψ39 was methylated by TrmL: the ribose methylation occurred at unmodified U34 [70]. Thus, the 5-carboxymethylaminomethyl group in cmnm^5^U34 is not involved in the recognition of tRNA by TrmL. Furthermore, the methylation of stem-loop RNA by TrmL [70] is consistent with the aforementioned proposed docking model of TrmL and tRNA [54].

### 4.4. Trm56

Trm56 catalyzes methylation of the ribose of C56 in tRNA [19,71]. Members of the Trm56 family have been found only in archaea. *Pyrococcus abyssi* Trm56 can methylate the ribose of C56 in stem-loop RNA corresponding to the T-arm [71]. Substitution of C56 by G abolished the methyl group acceptance activity of tRNA^Ser^, indicating that Trm56 recognizes the cytidine base at position 56 [71]. A crystal structure study of *Pyrococcus horikoshii* Trm56 revealed that positively charged amino acid residues are widely located around the catalytic pocket and the cleft of the dimer interface [53]. Therefore, the mode of tRNA binding cannot be predicted simply from the structure. A *P. horikoshii* Trm56 mutant enzyme, in which Glu111 (corresponding to Glu124 in motif 2 in *T. thermophilus* TrmH) was replaced with Ala, showed decreased, but still considerable methyltransfer activity, indicating that the Glu residue in motif 2 is not the catalytic center [53].

The product of *T. acidophilum Ta0931* gene is a unique member in the Trm56 family, because it has an additional HDPD-like domain at its C-terminal region (Figure 5) [43] (see Section 3).

It should be mentioned that the Cm56 modification in *Pyrobaculum aerophilum* tRNA is conferred by the C/D-box small RNA guide-dependent ribose methylation system [72,73] instead of Trm56 [72,73].

### 4.5. TrmD

TrmD catalyzes the transfer of methyl group from AdoMet to *N*^1^-atom of G37 in tRNA to form m^1^G37 [9]. In *E. coli*, only a subset tRNA with a G36G37 sequence possesses the m^1^G37 modification [33]. The tRNA selection mechanism by TrmD has been investigated extensively by many researchers. In 1997, Redlak et al. reported that TrmD can methylate a truncated tRNA, in which t- and d-arms have been deleted [74]. Foot-printing analysis of the TrmD-tRNA complex by chemical reagents showed that the anticodon-arm region is mainly protected [75]. In 2003, three groups reported the crystal structure of TrmD proteins and provided experimental evidences for the SPOUT superfamily [15,16,17]. Based on the TrmD-AdoHcy-phosphate ternary complex, a TrmD-tRNA docking model was constructed [15]. A structure-based site-directed mutagenesis study further elucidated both tRNA-binding site and important residues in the methyltransfer reaction [16]. Structural changes of anticodon-loop according to the formation of TrmD-tRNA complex have been investigated by chemical probing and fluorescent energy transfer experiments, which suggested that the G37 base is mobilized into the catalytic pocket only when the AdoMet-binding site is occupied by AdoMet or sinefungin (an analogue of AdoMet) [76]. We investigated the tRNA recognition mechanism of *A. aeolicus* TrmD and found that a micro-helix RNA corresponding to the anticodon-arm is the minimal substrate for this enzyme [77]. Unexpectedly, we found that *A. aeolicus* TrmD can methylate G37 in the A36G37 sequence, showing that purine36 is a positive determinant for *A. aeolicus* TrmD. Formation of a disulfide bond between the two subunits stabilizes the dimer structure of *A. aeolicus* TrmD and is required for enzymatic activity at high temperatures [78].

In eukaryotes and archaea, an m^1^G37 modification in tRNA is formed by Trm5, which has a class I fold catalytic domain [79,80,81]. Some archaeal Trm5 enzymes are involved in various methylation(s) ranging from m^1^G37 to wyosine derivatives [82,83,84], and eukaryote Trm5 methylates both cytoplasmic and mitochondrial tRNAs [85]. Because TrmD and Trm5 produce the same modified nucleoside at the same position in tRNA (m^1^G37), the enzymatic properties and tRNA recognition mechanisms of both enzymes have been compared [81,86,87]. Unlike TrmD, human [81] and archaeal [86,87] Trm5 require interaction with the t- and d-arms in tRNA for methylation. Furthermore, the 2-amino group in G37 is important for methylation by TrmD but it is dispensable for Trm5 [88]. Thus, the tRNA recognition mechanism of Trm5 is completely different from that of TrmD.

**Figure 7:**
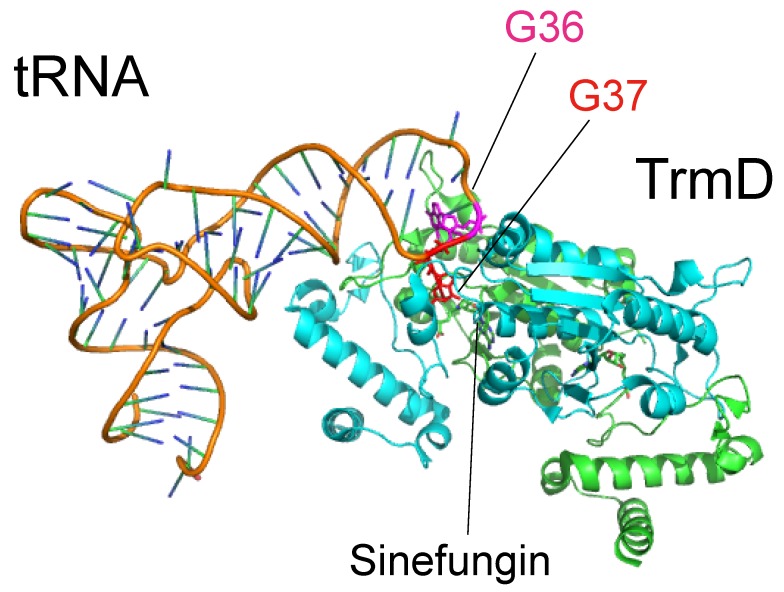
Structure of tRNA-TrmD-sinefungin ternary complex. The Protein Data Bank ID is 4yvi. TrmD and tRNA are shown by cartoon models. G36 (magenta) and G37 (red) bases in tRNA are highlighted by stick models. Two subunits of TrmD are colored in green and cyan.

In 2015, the crystal structure of the TrmD-tRNA-sinefungin ternary complex was reported (Figure 7) [89]. Structural comparison with the TrmD-AdoMet and TrmD-sinefungin binary complexes revealed that tRNA binding causes movement of the C-terminal region. The *N*^1^-atom of G37 in the bound tRNA forms a hydrogen bond with Asp169 residue and Arg154 is located near the G37 base. Replacement of Asp169 and Arg154 of *H. influenzae* TrmD with Ala causes a loss of activity, suggesting that these residues are involved in the catalytic mechanism [89], consistent with the results of a previous site-directed mutagenesis study of *E. coli* TrmD [16]. Furthermore, the additional crystal structures of ternary complexes, in which G36 in tRNA was replaced by U and C, were solved and an interesting hypothetical model was proposed in which G36 recognition occurs after G37 recognition but before the methyltransfer reaction [89]. Together, these crystal structures and the site-directed mutagenesis studies explain the selection of tRNA by TrmD [89].

### 4.6. TrmY

TrmY catalyzes the transfer of a methyl group from AdoMet to the *N*^1^-atom of Ψ54 and forms m^1^Ψ54 in tRNA [90]. The Ψ54 modification in tRNA is synthesized from U54 by archaeal Pus10 [90,91,92,93]. Members of TrmY family have been found only in archaea. TrmY from *Methanocaldococcus jannaschii* can modify t-arm-like micro-helix RNA, and the neighboring pyrimidine at position 55 is a positive determinant for the enzyme [90].

### 4.7. Trm10

Trm10 from *S. cerevisiae* catalyzes methylation of the *N*^1^-atom of G9 to form m^1^G9 in tRNA [94]. The crystal structures of Trm10 from *S. cerevisiae* and *Schizosaccharomyces pombe* have been reported [49]. Although the catalytic domains of both Trm10 proteins showed the typical SPOUT fold, X–ray scattering analysis revealed that Trm10 behaves as a monomer in solution [49]. In most SPOUT tRNA methyltransferases, the catalytic pocket is formed by two subunits. However, the crystal structure and structure-based site-directed mutagenesis study revealed that the catalytic pocket of Trm10 is formed in one subunit and that the C-terminal region is involved in the tRNA recognition [49].

So far, the region(s) in tRNA recognized by Trm10 have not been clarified. Swinehart et al. have reported the in vitro methylation of tRNAs, which are not methylated in vivo, by Trm10 [95]. Furthermore, the overexpression of Trm10 in yeast leads to m^1^G9 modification in tRNAs that are normally unmodified. Thus, the m^1^G9 modification in yeast tRNA seems to be regulated by the relative amounts of substrate tRNA and Trm10.

In human mitochondria, a homolog of Trm10 (TRMT10C) is a subunit of RNase P and catalyzes the *N*^1^-methylation of purine at position 9 in tRNA [96]. Because this modification pattern resembles that of archaeal Trm10, it will be interesting to determine the catalytic mechanism of TRMT10C.

### 4.8. aTrm10

*Sulfolobus acidocaldarius* Trm10 forms m^1^A9 in tRNA, whereas *Thermococcus kodakarensis* Trm10 forms m^1^A9 and m^1^G9 in tRNA [97]. Multi-angle light scatter and small angle X–ray scatter experiments revealed that *S. acidocaldarius* Trm10 is a monomeric enzyme like eukaryotic Trm10 [30]. The crystal structure study of *S. acidocaldarius* Trm10 revealed the presence of an N-terminal domain that is not observed in other tRNA methyltransferases with a SPOUT fold (Figure 5) [30]. Based on the crystal structure, a site-directed mutagenesis study was performed and an aTrm10-tRNA docking model was constructed [30]. The docking model suggested that the anticodon-arm and local structure around the target site (A9) in tRNA are likely to be recognized by aTrm10 [30].

## 5. Functions of Modified Nucleosides Synthesized by SPOUT tRNA Methyltransferases

Transfer RNA is an adaptor molecule that enables the genetic code of nucleic acids to be converted to amino acids in protein. Consequently, the primary function of an individual tRNA modification is linked to the different steps of protein synthesis. For a long time, modifications in the three-dimensional core in tRNA were considered to contribute stabilization of the l-shaped tRNA structure, however, recent studies are gradually elucidating the functions of modified nucleosides beyond the structural role. In this section, the functions of modified nucleosides, which are synthesized by SPOUT tRNA methyltransferases, are summarized. Furthermore, the relationship of modified nucleoside and its associated enzyme to higher biological phenomena, and their potential utilization in drug design and therapy are described.

### 5.1. Gm18

Gm18 modification is often observed in eubacteria and eukaryote tRNAs [33], where it is produced by TrmH [7] and Trm3 [47], respectively. However, the function of Gm18 modification in tRNA has been unclear for a long time. An early study indicated that Gm18 modification protects the d-loop region from RNase T1 digestion; therefore, it was considered that it might contribute to prolonging the half-life of tRNA [8]. However, an *E. coli trmH* gene disruptant strain did not show growth delay in the rich or minimal 3-(*N*-morpholino)-propane-sulfonic acid (MOPS)/glucose medium at 37 °C or 42 °C [7]. Furthermore, a *S. cerevisiae trm3* gene disruptant strain did not exhibit any growth delay when grown at various temperatures (37 °C, 30 °C or 19 °C) in rich medium, glucose-lacking medium, minimum medium, or nonfermentable medium [47]. In 2002, it was reported that a combination of *trmH*, *trmA* and *truB* mutations in *E. coli* reduced the growth rate [98]: TrmA [99] and TruB [100] are tRNA (m^5^U54) methyltransferase and tRNA (Ψ55) synthase, respectively. The Gm18 (by TrmH), m^5^U54 (by TrmA) and Ψ55 (by TruB) modifications are assembled at the elbow region of tRNA and stabilize the l-shaped tRNA structure (the formation of tertiary base pairs and stabilization of l-shaped tRNA structure by modified nucleotides are reviewed in reference [101]). Thus, Gm18 modification works cooperatively with other modifications in tRNA [98].

In the case of *T. thermophilus*, an extremely thermophilic eubacterium, Gm18 modification functions in the network between the modified nucleotides and tRNA modification enzymes. In brief, *T. thermophilus* can grow at a wide range of temperatures (50−83 °C). To facilitate this, a network in *T. thermophilus* regulates the modification levels of Gm18, 5-methyl-2-thiouridine at position 54 (m^5^s^2^U54) [102,103,104,105,106] and m^1^A58 [107,108] in tRNA, controls the flexibility (rigidity) of tRNA, and contributes to efficient protein synthesis at various temperatures [109,110,111]. Although the Gm18 modification itself increases the melting temperature of tRNA^Phe^ by only 0.5 °C, the combination of these three modified nucleosides increases the melting temperature by nearly 10 °C [110]. Thus, in thermophilic bacteria, Gm18 contributes to regulate the flexibility (rigidity) of tRNA at various temperatures.

**Figure 8:**
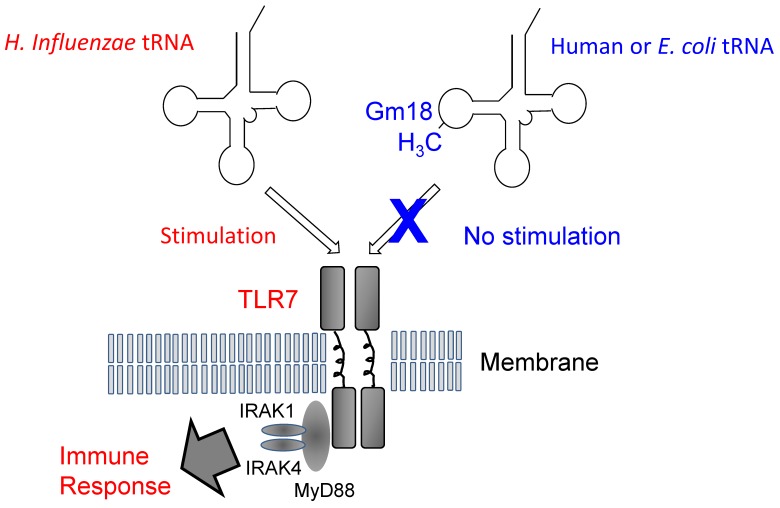
Model of the immune response and Gm18 methylation in tRNA. Transfer RNA from *H. influenzae*, a respiratory infectious bacterium, induces dimer formation by Toll like receptor-7 (TLR7), the immune response is then stimulated via binding of the proteins, MyD88, IRAK1 and IRAK4. In contrast, human and *E. coli* tRNAs do not stimulate TLR7 because they contain the Gm18 modification. The *E. coli* *trmH* gene disruptant strain does not show any obvious phenotype under laboratory culture conditions [7]. Both Gm18 modification and TrmH are required for survival of *E. coli* in animal gut.

In 2012, new progress on the physiological role of Gm18 modification was reported. When an exogenous single-stranded RNA such as *H. influenzae* tRNA is present in humans, Toll-like receptor 7 (TLR7) forms a dimer structure and then activates the immune response system (Figure 8). However, endogenous or *E. coli* tRNA does not stimulate TLR7. The mechanism of differentiation between exogenous and endogenous tRNA has been independently clarified by two groups, who found that the Gm18 modification in *E. coli* tRNA suppresses immunostimulation via TLR7 [112,113]. Thus, enterobacteria exploit the Gm18 modification in tRNA to avoid the host immune system. Given that Gm18-modified tRNA acts as an antagonist of TLR7, Gm18-modified tRNA might be used as an anti-inflammatory drug [113]. Moreover, the Gm18 modification in human tRNA functions as a marker of endogenous tRNA.

### 5.2. Nm32

For a long time, 2’-*O*-methylation of ribose at position 32 in tRNA was considered to stabilize the anticodon-loop structure.

During preparation of this manuscript, however, it was reported that *P. aeruginosa* TrmJ is required for resistance to oxidative stress [66]. A decrease in the expression of catalases and regulatory transcription factor *oxyR* genes is observed in a *P. aeruginosa* TrmJ mutant strain under H_2_O_2_ stress [66]. Therefore, the Nm32 modification catalyzed by TrmJ may be required for the sufficient expression of transcription factors such as OxyR. Given that *P. aeruginosa* is an opportunistic pathogen, TrmJ has the potential to be a target protein for drug design [66].

In eukaryotes, the 2’-*O*-methylation of ribose at position 32 is catalyzed by the Trm7-Trm732 complex [114,115]. Trm7 is a class I AdoMet-dependent tRNA methyltransferase [114]. The site specificity of Trm7 is regulated by the existence of a partner subunit (Trm732 or Trm734) [115,116]. The Cm32 modification in yeast tRNA^Phe^ is required for the formation of wybutosine at position 37 from m^1^G37 [115,117]. The human counterpart of Trm7 is FTSJ1, in which mutations cause nonsyndromic X-linked mental retardation [118,119,120].

### 5.3. Cm34 and cmnm^5^Um34

Position 34 is the first letter of the anticodon in tRNA. Therefore, modifications at position 34 have direct effects on decoding processes on the ribosome, such as codon-anticodon interaction and prevention of frame-shift errors [121,122]. Given that the 2’-*O*-methyl group in UmpU stabilizes the C3’-endo form of ribose [123], the 2’-*O*-methylation by TrmL probably stabilizes the conformation of anticodon. Transfer RNAs with 2’-*O*-methylation at position 34 are used for the separation of a four-codon box to two two-codon boxes [124].

In eukaryotes, the 2’-*O*-methylation of ribose at position 34 is produced by the Trm7-Trm734 complex [114,115,116,117]. Therefore, similar to TrmJ, TrmL might have potential as a target protein for drug design.

### 5.4. Cm56

Cm56 modification in tRNA is observed only in archaea [33]. As described above, 2’-*O*-methylation shifts the equilibrium of ribose puckering to the C3’-endo form [123]; therefore, the Cm56 modification may stabilize the G19-Cm56 tertiary base pair in tRNA.

### 5.5. m^1^G37

Because m^1^G37 modification prevents a frame shift error during protein synthesis [125,126,127] and accelerates the Elongation Factor Thermo unstable (EF-Tu) -dependent A-site binding of tRNA^Pro^ and tRNA^Arg^ [128], m^1^G37 and TrmD are essential for the viability of several eubacteria [125,129,130]. In eukaryotes and archaea, the m^1^G37 modification in tRNA is produced by Trm5 [81,85]. The enzymatic properties of TrmD and Trm5 are considerably different from each other [81,86,87]. Therefore, TrmD is an important target protein for design of antibacterial drugs [131]. Indeed, selective inhibitors for TrmD have been reported [132].

### 5.6. m^1^Ψ54

The function of m^1^Ψ54 has not been reported. However, the structural similarity between m^1^Ψ54 and m^5^U54 (Figure 1) suggests that an m^1^Ψ54-A58 (or m^1^A58 [133]) tertiary base pair is formed in the t-loop to stabilize the l-shaped tRNA structure.

### 5.7. m^1^G9 and m^1^A9

In the crystal structure of *S. cerevisiae* tRNA^Phe^, there is an A9-A23-U12 tertiary base pair. The methyl group in m^1^G9 or m^1^A9 does not structurally hinder the formation of this tertiary base pair. Given that m^1^G and m^1^A prevent the formation of a Watson-Crick base pair, m^1^G9 and m^1^A9 may support the formation of m^1^G9 (m^1^A9)-A23-U12 tertiary base pair and result in stabilization of the l-shaped tRNA structure.

Three Trm10 homologs are encoded in the human genome [94]. Deficiency in one of them TRMT10A, causes young onset diabetes [134,135]. Defects in other tRNA modification enzyme, Cdk5 regulatory associated protein 1-like 1, a tRNA modification enzyme involved in the biosynthesis of 2-methylthio-*N*^6^-threonylcarbamoyladenosine at position 37 (ms^2^t^6^A37) causes type 2 diabetes [136]. Therefore, some tRNA modifications seem to be related to diabetes and the associated genes might be utilized for genetic diagnosis and might have potential as targets of gene therapy.

In human mitochondria, modified nucleosides including m^1^A9 in tRNA^Lys^ are required for cloverleaf folding [137]. In nematode mitochondrial t-arm-less tRNAs, m^1^A9 modification is required for effective aminoacylation and EF-Tu binding [138].

Although the phenomena might be not caused by defects in tRNA modification, following findings have been reported. The expression level of HRG9MTD2 (one of human Trm10 paralogs) is changed in colorectal cancer [139,140]. Recessive mutations in human TRMET10C, a subunit of human mitochondrial RNase P [96], cause defects in mitochondrial RNA processing and multiple respiratory chain deficiencies [141]. In *Drosophila*, loss of the mitochondrial RNase P subunit is lethal [142].

## 6. Perspective

For a decade, much data on tRNA methyltransferases with a SPOUT fold have been accumulated. The target RNA molecules and resulting modified nucleosides of many candidate proteins, which were predicted by bioinformatics studies as RNA methyltransferases with a SPOUT fold [2,3,29], have been identified. Even today, however, it is difficult to predict the target RNA molecule and the position modified in RNA from the amino acid sequence of a candidate protein. Therefore, a combination of bioinformatics study and biochemical analysis will be necessary to obtain these data in the future. In the genome of multicellular organisms such as human [143], there are multiple homologs of putative tRNA methyltransferase genes. The products of these genes seem to share the functions in the organism. As a result, studies on gene knock out strains will be also necessary to clarify the function of each candidate protein.

Our understanding of the catalytic mechanisms and functions of knot structures of SPOUT RNA methyltransferases has been also deepened. Nevertheless, many enigmas remain. For example, the mechanism by which methylated tRNA and AdoHcy are released after the methyltransfer reaction is not clear. For complete understanding of the reaction mechanism, further structural and biochemical studies will be required.

It is becoming clear that the modified nucleosides in tRNA and associated tRNA modification enzymes are involved in higher biological phenomena beyond their roles in protein synthesis. In some cases, a defect in a tRNA modification enzyme gene causes genetic disease [140]. Therefore, studies on the tRNA modification enzyme genes and modified nucleosides are directly linked to our understanding of the molecular mechanism of genetic diseases and the development of genetic diagnosis and gene therapy.

In the case of eukaryotes, tRNA methylations work coordinately as stabilizing factors and markers of maturation, and the degree of modification changes in response to various stresses. In some cases, hypomodified tRNAs are degraded aggressively. For example, in the *S. cerevisiae* *trm4* (synthesizes m^5^C at multiple sites) [144] and *trm8* (produces m^7^G46) [145] double knock-out strain, the half-life of tRNA^Val^ is shortened and the strain shows a growth defect [146]. Therefore, tRNA modifications stabilize tRNA structure coordinately and systems to degrade hypomodified tRNAs exist in eukaryotic cells [146,147,148]. Furthermore, in *S. cerevisiae,* the m^1^A58 modification by the Trm6–Trm61 complex regulates both the degradation of initiator tRNA^Met^ and its transport from the nucleus to the cytoplasm [149,150,151]. The m^1^A58 modification functions as a marker of maturation and absence of modification leads to degradation of initiator tRNA^Met^. Thus, m^1^A58 is part of the RNA quality control system. Moreover, in the case of *S. cerevisiae,* splicing is performed in the cytoplasm [152] and precursor tRNAs are matured during repeated-transports between the nucleus and cytoplasm [153]. Therefore, some tRNA modifications might act as the markers of maturation at halfway checkpoints. In addition, several modifications in tRNA from eukaryotes are identified as stress-response (or stress-tolerance) factors [154,155,156,157]. In the future, the modified nucleoside(s), which is formed by a SPOUT tRNA methyltransferase, may be found to be related to these biological phenomena in eukaryotic cells.

There are many cases in which the same modification at the same position in tRNA is produced by different enzymes in eukaryotes and bacteria, for instance, Trm5 and TrmD, and Trm7 and TrmJ (see Section 5). There are many additional examples, although the responsible enzymes are not all members of SPOUT tRNA methyltransferase superfamily. For example, the m^1^A58 modification is produced by the Trm6-Trn61 complex in eukaryotic tRNA [149,150], whereas it is produced by TrmI [107,108] and archaeal TrmI [133] in eubacterial and archaeal tRNA, respectively. Furthermore, m^5^U54 modification is produced by Trm2 in eukaryotic tRNA [158], whereas it is produced by TrmA in eubacterial tRNA [99] and some archaeal tRNA [159] and a folate-dependent methyltransferase, TrmFO [104,106,160]. Consequently, these enzymes in infectious bacteria have potential as targets for the design of antibacterial drugs. Thus, studies on bacterial tRNA modification enzymes and modified nucleosides in tRNA are also important. Moreover, infection by retro-virus is strongly related to tRNA modification enzymes and modified nucleosides in tRNA. For example, human immunodeficiency virus (HIV) utilizes the m^1^A58 modification in tRNA^Lys^3 as the termination factor of reverse transcription [161] and requires TARBP1 (human Trm3 homolog) for binding of trans-activating response (TAR) RNA to RNA polymerase II [162]. Therefore, studies on tRNA modification enzymes and modified nucleosides in tRNA will contribute to both our understanding of the molecular mechanism underlying viral infection and the design of anti-viral drugs. In addition, Gm18-modified tRNA might be used as an anti-inflammatory drug [113]. Thus, the importance of studies on tRNA modification enzymes and modified nucleosides in tRNA is increasing in the medical and pharmaceutical sciences.

RNA modification enzymes and modified nucleosides in RNA are continuing to evolve even today. The most powerful driving force for evolution is the existence of infectious organisms. Hosts require systems to discriminate between endogenous and exogenous RNAs to prevent infection, while infectious organisms need to avoid the defense systems of hosts to survive. Thus, studies on tRNA modification enzymes and modified nucleosides in tRNA will continue to be necessary while infectious organisms continue to exist.

## Supplementary Materials

Supplementary File 1
